# Long-Term, CD4^+^ Memory T Cell Response to SARS-CoV-2

**DOI:** 10.3389/fimmu.2022.800070

**Published:** 2022-04-20

**Authors:** Sebastian Wirsching, Laura Harder, Markus Heymanns, Britta Gröndahl, Katja Hilbert, Frank Kowalzik, Claudius Meyer, Stephan Gehring

**Affiliations:** Children’s Hospital, University Medical Center of the Johannes Gutenberg University, Mainz, Germany

**Keywords:** SARS-CoV-2, HCoV, T cell immunity, long-term memory, cross-reactivity

## Abstract

The first cases of coronavirus disease-19 (COVID-19) caused by severe acute respiratory syndrome coronavirus 2 (SARS-CoV-2) were reported by Chinese authorities at the end of 2019. The disease spread quickly and was declared a global pandemic shortly thereafter. To respond effectively to infection and prevent viral spread, it is important to delineate the factors that affect protective immunity. Herein, a cohort of convalescent healthcare workers was recruited and their immune responses were studied over a period of 3 to 9 months following the onset of symptoms. A cross-reactive T cell response to SARS-CoV-2 and endemic coronaviruses, i.e., OC43 and NL63, was demonstrated in the infected, convalescent cohort, as well as a cohort composed of unexposed individuals. The convalescent cohort, however, displayed an increased number of SARS-CoV-2-specific CD4^+^ T cells relative to the unexposed group. Moreover, unlike humoral immunity and quickly decreasing antibody titers, T cell immunity in convalescent individuals was maintained and stable throughout the study period. This study also suggests that, based on the higher CD4 T cell memory response against nucleocapsid antigen, future vaccine designs may include nucleocapsid as an additional antigen along with the spike protein.

## Introduction

On December 31, 2019, authorities in Wuhan, China reported a cluster of atypical pneumonia cases (1). The illness, caused by severe acute respiratory syndrome coronavirus 2 (SARS-CoV-2), was named coronavirus disease 19 (COVID-19) shortly thereafter (2). Approximately 80% of infections are asymptomatic or cause only mild symptoms such as a dry cough, dyspnea and fever. Fifteen percent of cases require supplemental oxygen, however, while 5% develop acute respiratory distress syndrome (ARDS) and need active ventilation (3, 4). The virus spread globally in a matter of weeks leading the WHO to declare a pandemic in March 2020 (1). Over 200 million cases were identified and nearly almost 5 million COVID-19-related deaths occurred worldwide by October, 2021 (5). Early analysis of samples obtained from active, as well as convalescent, SARS-CoV-2-infected individuals demonstrated humoral and cell-mediated immune responses, and a correlation between antibody titers and T cell reactivity (6–8).

Recent reports indicate that neutralizing antibody titers decrease significantly over the 8-month period following symptom onset, and decrease up to 53% after a year (9, 10). Reports of individuals who contracted COVID-19 a second time within a year emphasize the import of this finding (11, 12). A better understanding of the dynamics of reinfections would benefit greatly from an in-depth study of the development of cellular immunity. In this regard, other investigators reported that SARS-CoV-2-specific T cell responses were stable for 8 to 10 months (13, 14). Vaccines approved currently only target the spike (S) glycoprotein and its receptor binding domain (RBD) used to gain entrance into host cells (15–18). Notably, convalescent patients exhibit T cell response to additional structural proteins, i.e., envelope (E), membrane (M) and nucleocapsid (N) (8). Incorporation of these other targets into next generation vaccines could significantly improve protection.

A cohort of convalescent healthcare workers was recruited and studied for a 9-month period. T cell responses to three SARS-CoV-2-associated antigens (S, M and N), as well as to the endemic coronaviruses HCoV-OC43 and HCoV-NL63, were determined. Individuals previously exposed to SARS-CoV-2 exhibited a greater T cell response than did unexposed people. Convalescent individuals also clearly showed cross-reactivity between SARS-CoV-2 and HCoV. Importantly, in contrast to diminishing antibody titers, stable anti-SARS-CoV-2-specific T cell responses were observed throughout the 9-month study period.

## Material and methods

### Human Subjects

Convalescent COVID-19 patients were diagnosed by RT-PCR or SARS-CoV-2-specific antibody detection. Antibody screening was conducted throughout April and May 2020. Subjects who provided proof of a positive RT-PCR test or ≥3 positive serological tests using the two assays described immediately below were deemed SARS-CoV-2-positive. Positive PCR tests had a median date of March 17, 2020. Blood was collected between June 2020 and December 2020 at three distinct time points: at approximately 3 (Visit 1, V1), 6 (Visit 2, V2), and 9 (Visit 3, V3) months following the onset of symptoms. An unexposed healthy control group was matched by age, gender and their clinical department. Main criterium for inclusion into the unexposed control group was no detectable SARS-CoV-2 antibody titer across all three visits. Blood collection was approved by the local ethics committee (Ethik-Kommission der Landesärztekammer Rheinland-Pfalz) (No. 2020-14968). All individuals enrolled in the study provided informed consent.

### SARS-CoV-2 Serology

All serological tests were performed using both the Abbott ARCHITECT SARS-CoV-2 IgG Assay (Abbott Laboratories, Abbott Park, IL, USA), an automated two-step immunoassay using SARS-CoV-2 antigen-coated paramagnetic microparticles, and the Roche Elecsys^®^ Anti-SARS-CoV-2 Assay (Roche Diagnostics GmbH – Mannheim, Germany), an electro-chemiluminescence immunoassay that quantifies total SARS-CoV-2-specific immunoglobulin (19). The Abbott ARCHITECT SARS-CoV-2 IgG Assay specifically measures anti-SARS-CoV-2 IgG antibodies whereas the Roche Elecsys^®^ Anti-SARS-CoV-2 Assay measures total anti-SARS-CoV-2 antibody, resulting in ~35-fold higher antibody titers in the Roche assay.

### Cell Isolation and Culture

Peripheral blood mononuclear cells (PBMCs) were isolated from heparinized whole blood by Biocoll density gradient centrifugation in SepMate tubes (StemCell Technologies, Vancouver, BC, Canada). Isolated cells were washed twice with Hank’s Balanced Salt Solution, frozen overnight at -80°C, then moved and stored in liquid nitrogen until use. The cells were thawed, suspended in *X-VIVO* 15 serum-free medium (Lonza Biologics) supplemented with 1% penicillin/streptomycin and inoculated into 96-well tissue culture plates at 1 x 10^6^ cells per well. Afterwards, cells were stimulated with either of the following peptide pools: SARS-CoV-2 spike glycoprotein (PM-WCPV-S-1, two peptide pools composed of overlapping spike glycoprotein sequences; pool S-1, peptide sequences spanning the N-terminus and pool S-2, peptide sequences spanning the C-terminus), SARS-CoV-2 NCAP (PM-WCPV-NCAP-1, peptide pool composed of overlapping nucleocapsid sequences), SARS-CoV-2 VME1 (PM-WCPV-VME-1, peptide pool composed of overlapping membrane protein sequences), HCoV-OC43 spike glycoprotein (PM-OC43-S-1, two peptide pools composed of overlapping spike glycoprotein sequences; pool OC43-1, peptide sequences spanning the N-terminus and pool OC43-2, peptide sequences spanning the C-terminus) and HCoV-NL63 spike glycoprotein (PM-NL63-S-1, two peptide pools composed of overlapping spike glycoprotein sequences; pool NL63-1 peptide sequence spanning the N-terminus and pool NL63-2 peptide sequences spanning the C-terminus). All peptide pools, synthesized as 15mers with 11 amino acid overlaps, were purchased from JPT Peptide Technologies GmbH, Berlin, Germany. Pools were dissolved in DMSO and added to cultures at 1 µg/ml per peptide according to the manufacturer’s instructions. Negative controls consisted of cells incubated with DMSO only. Cells stimulated with 1.5 µg/ml Staphylococcal enterotoxin B (SEB) served as a positive control. Additionally, unconjugated anti-CD28 (clone CD28.2) and anti-CD49d (clone 9F10) monoclonal antibodies (0.5 µg/ml each; BD Biosciences, Franklin, Lakes, NJ, USA) were added to provide co-stimulation. Cells were incubated for 16 hours at 37°C and 5% CO_2_. Brefeldin A (BioLegend, San Diego, CA, USA) was added after the first two hours incubation to inhibit secretion.

### Flow Cytometry

Stimulated cells were transferred to FACS tubes and washed. The cells were stained extracellularly for 10 min at room temperature with Viobility 405/452 Fixable Dye, antibodies against CD14, CD20, both conjugated to VioBlue (Miltenyi Biotec, Bergisch Gladbach, Germany) and CD4 conjugated to PerCP (BD Biosciences). Subsequently, the stained cells were permeabilized using the BD Cytofix/Cytoperm kit (BD Biosciences) according to manufacturer’s instructions. Permeabilized cells were then stained intracellularly for 10 min at room temperature with antibodies against IL-2 conjugated to BV605 (BD Biosciences), TNF- α conjugated to PE-Vio 770, CD154 conjugated to PerCP-Vio 700, and IFN-γ conjugated to APC-Vio770 (Miltenyi Biotec) according to the methods of the supplier. Stained cells were quantified with a MACSQuant16^®^ analyzer flow cytometer (Miltenyi Biotec). FACS data were evaluated using FlowLogic software version 8.4 (Inivai Technologies, Victoria 3194 Australia). Reactive T cells were defined as CD4^+^ T cells expressing ≥2 of the following T_H1_ activation markers: CD154, IFN-γ, IL-2 and TNF-α. DMSO background controls were subtracted from the data shown. The gating strategy used for all analyses can be found in the supplementary material (**Figures S1** and **S2**).

### Statistical Analysis

Statistical analysis was performed using GraphPad Prism version 7. FACS data were analyzed using a non-paired two-tailed Mann-Whitney-U tests; a Friedman test was used to analyze the serological data. Data was deemed significant if it passed a *P*<0.05.

## Results

### Study Cohort

Thirty-six convalescent SARS-CoV-2 patients with a mean age of 35.8 years were included in the study; 69.4% were female (**Table S1**). An unexposed control group was matched both in age (37.1 years) and sex (69.4% female). SARS-CoV-2 infection was verified by PCR test in 63.9% of the convalescent group; 94.4% of the individuals showed a positive anti-SARS-CoV-2 antibody titer. All convalescent subjects experienced a mild infection. The most frequently reported symptoms (64%) were loss of taste or smell and headache. Other commonly reported symptoms included a dry cough (44%) and fever (42%). Sixteen percent of individuals reported no symptoms albeit SARS-CoV-2 infection was verified by PCR or antibody testing. One subject had no detectable serum antibodies throughout the course of the study although infection was verified by PCR.

### Convalescent Individuals Exhibit Broad Reactivity Against SARS-CoV-2

**Figure 1:**
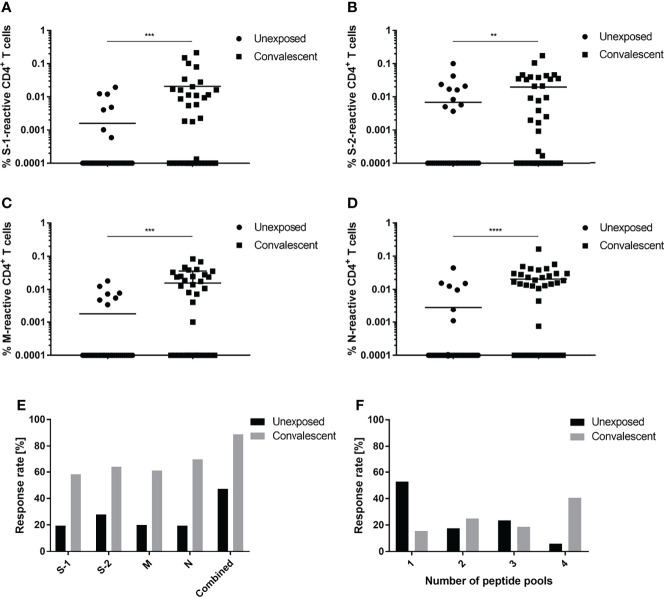
CD4^+^ T cell response specific for SARS-CoV-2. T cell responses of unexposed (circles) and convalescent (squares) individuals against S-1 **(A)**, S-2 **(B)**, M **(C)** or N **(D)** SARS-CoV-2 peptide pools. **(E)** Proportional CD4^+^ T cell response rates of unexposed (black) and convalescent (grey) study participants to individual (S-1, S-2, M, N) or a combination of all four (combined) peptide pools. **(F)** Proportional distribution of study participants reacting to one or an aggregate of more than one peptide pool. Reactive T cells were defined as CD4^+^ T cells expressing ≥2 T_H1_ activation markers (CD154, IFN-γ, IL-2, TNF-α). DMSO background controls were subtracted from the data shown. Data are shown with the means. **(A–E)** n = 36; **(F)** Unexposed n = 17, convalescent n = 32. Statistically different **(A–D)**: **P < 0.01, ***P < 0.001, ****P < 0.0001 (non-parametric two-tailed Mann-Whitney-U test).

Initial experiments were undertaken to determine and compare the percentages of SARS-CoV-2-specific T cells among the PBMCs obtained from convalescent and unexposed individuals at 3 months post symptom onset. Convalescent individuals showed a greater frequency of CD4^+^ T cells specific for the N-terminal (S-1) and C-terminal (S-2) spike glycoprotein peptide pools, as well as for the membrane (M) and nucleocapsid (N) peptide pools (**Figures 1A–D**). Of these individuals, 88.9% exhibited a response toward at least one of the peptide pools (**Figure 1E**). Both the S-1 and S-2 pools were recognized by approximately 60% of individuals; 61.8% of individuals recognized the M peptide pool. The strongest and most frequent response exhibited by 69.44% of convalescent individuals was specific for N. Of the individuals in the control group, 47.2% recognized at least one of the SARS-CoV-2 antigen pools tested. Responses of the control group to S-1 and S-2 were 19.4% and 27.8%, respectively. Additionally, a greater number of convalescent patients recognized ≥2 SARS-CoV-2 peptide pools (**Figure 1F**). Of the 32 reactive study participants, 15.6% responded to only one, while a greater percentage of individuals in the convalescent group recognized two (25%), three (18.7%) or four (40.6%) peptide pools. Comparison of T cell responses against SARS-CoV-2 peptide pools of individual convalescent donors revealed that T cell frequencies are very dependent on the respective individual. Some donors, e.g., 32, 33 and 34, showed very similar responses against all four antigens, whereas others, e.g., donors 2, 8 and 11, react differently against each antigen. (**Figure S3**). Analysis of CD154^+^/IFN-γ^+^, CD154^+^/IL-2^+^ and CD154^+^/TNF-α^+^ SARS-CoV-2-reactive CD4^+^ T cells revealed similar frequencies of antigen-specific T cells (**Figure S4**).

### SARS-CoV-2 and Endemic Coronaviruses Spike Glycoproteins Exhibit Strong Cross-Reactivity

**Figure 2:**
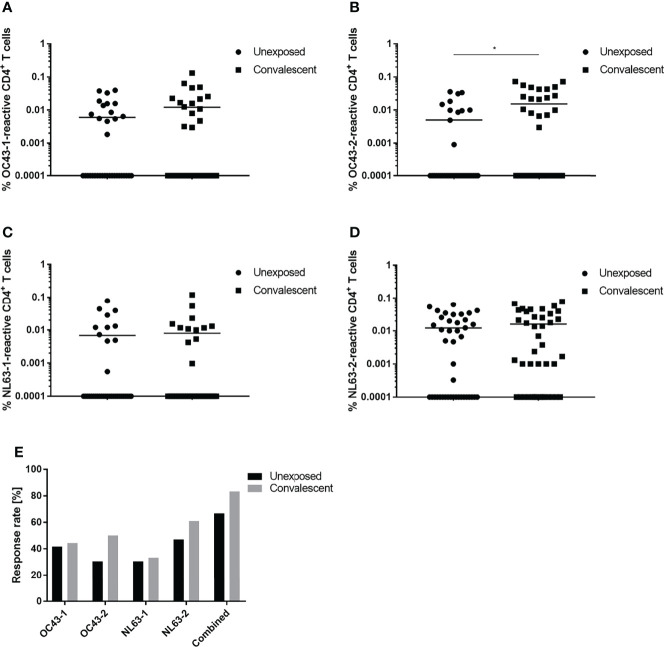
Response of CD4^+^ T cells to endemic HCoV peptide pools. Comparison of T cell responses of unexposed (circles) and SARS-CoV-2 convalescent (squares) study participants to OC43-1 **(A)**, OC43-2 **(B)**, NL63-1 **(C)** and NL63-2 **(D)** HCoV spike peptide pools. **(E)** Comparison of the proportional CD4^+^ T cell response rate of unexposed (black) and convalescent (grey) donors to individual or a combination of all four (combined) HCoV peptide pools. Reactive T cells were defined as CD4^+^ T cells expressing ≥2 T_H1_ activation markers (CD154, IFN-γ, IL-2, TNF-α). DMSO background controls were subtracted from the data shown. Data are shown with the means (n = 36). Statistically different: *P < 0.05 (non-parametric two-tailed Mann-Whitney-U test).

Cross-reactivity between the spike glycoproteins expressed by SARS-CoV-2 and the endemic coronaviruses, HCoV-OC43 and HCoV-NL63, was examined. A comparison of the SARS-CoV-2-convalescent and unexposed groups showed that the percentage of reactive CD4^+^ T cells specific for the HCoV-OC43 and HCoV-NL63 spike glycoprotein peptide pools was very similar (**Figures 2A–D**). Eighty-three percent of the convalescent subjects recognized at least one of the four HCoV peptide pools tested compared to only 66.7% of the unexposed subjects (**Figure 2E**). Interestingly, convalescent individuals tended to respond slightly more frequently to the peptide pools that comprise the two C-terminal peptide sequences, OC-43-2 (50%) and NL63-2 (61.1%), than did the unexposed individuals (i.e., 30.6% and 47.2% respectively). Specific responses to the N-terminal peptide pools (OC-43-1 and NL-63-1) occurred in 30-40% of the study participants enrolled in both groups.

**Figure 3:**
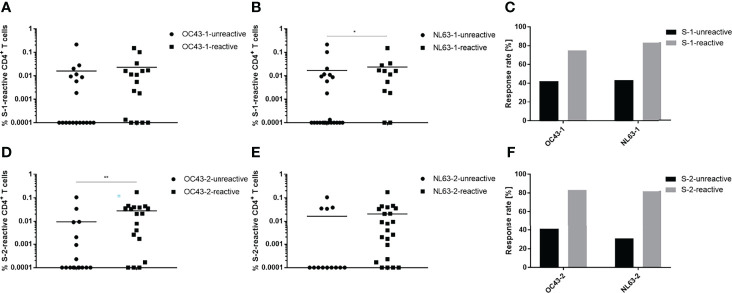
SARS-CoV-2 spike protein-specific T cells recognize endemic HCoV spike proteins. Convalescent individuals unreactive (circles) or reactive (squares) against OC43 and NL63 N-terminal **(A, B)** or C-terminal **(D, E)** HCoV peptide pools were analyzed for their responses toward their respective SARS-CoV-2 counterpart. Comparison of the proportional response rates of HCoV unreactive (black) and reactive (grey) individuals toward SARS-CoV-2 spike glycoprotein S-1 **(C)** and S-2 **(F)** peptide pools. Reactive T cells were defined as CD4^+^ T cells expressing at ≥2 T_H1_ markers (CD154, IFN-γ, IL-2, TNF-α). DMSO background controls were subtracted from the data shown. Data are shown with the means. **(A)** unreactive n = 19, reactive n = 16; **(B)** unreactive n = 23, reactive n = 12; **(D)** unreactive n = 17, reactive n = 18; **(E)** unreactive n = 13, reactive n = 22. Statistically different **(A, B, D, E)**: *P < 0.05, **P < 0.01 (non-parametric two-tailed Mann-Whitney-U test).

To analyze cross-reactivity between the HCoV and SARS-CoV-2 spike glycoproteins, the convalescent group was divided into subgroups reactive or unreactive against either the N- or C-terminus of OC43 and NL63; the percentages of SARS-CoV-2 S-1- and S-2-specific T cells were then determined (**Figure 3**). Study participants that reacted against OC43-1 and NL63-1 also possessed a slightly higher percentage of S-1-specific T cells (**Figures 3A, B**). Moreover, those participants exhibited an approximately two-fold higher response rate than the HCoV-unreactive group (**Figure 3C**). The OC43-2-reactive subgroup demonstrated a similar effect, i.e., a significantly larger percentage of S-2-specific T cells (**Figure 3D**). NL63-2-reactive T cells, on the other hand, showed only a marginal difference compared to the unreactive group (**Figure 3E**). Both C-terminal groups, however, showed similar increases in response rate that were greater than the N-terminal groups (**Figure 3F**). OC43-2-reactive subjects showed an approximately two-fold increase in overall response rate whereas NL63-2-reactive subjects exhibited nearly a three-fold increase. Notably, the unexposed group displayed similar trends in its T cell response rates demonstrating a greater response to peptide pools composing the C terminus, compared to the N terminus, of the HCoV spike glycoprotein (**Figure S5**).

### T Cell Anti-SARS-CoV-2 Reactivity Remains Stable for at Least 9 Months

**Figure 4:**
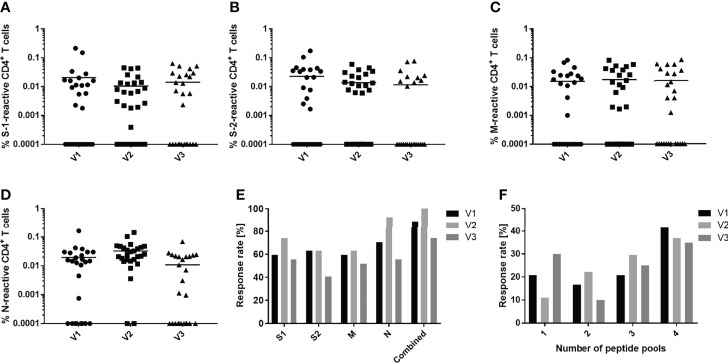
Development of peptide-specific CD4^+^ T cell responses. Comparison of immune responses of convalescent individuals against S-1 **(A)**, S-2 **(B)**, M **(C)** and N **(D)** SARS-CoV-2 peptide pools over the course of a 9-month period. Peripheral blood was drawn at three timepoints: 3 months (V1, circles), 6 months (V2, squares) and 9 months (V3, triangles) post infection and symptom onset. **(E)** Proportional CD4^+^ T cell response rate of convalescent donors to individual (S-1, S-2, M, N) or a combination of all four (combined) peptide pools. **(F)** Proportion of convalescent donors that respond to one or an aggregate of more than one peptide pool during the period indicated. Reactive T cells were defined as CD4^+^ T cells expressing ≥2 T_H1_ markers (CD154, IFN-γ, IL-2, TNF-α). DMSO background controls were subtracted from the data shown. Data are shown with means **(A–E)** n = 27; **(F)** V1 n = 24, V2 n = 27, V3 n = 20.

Experiments were undertaken to determine and compare the longevities of the cellular and humoral responses of convalescent patients to SARS-CoV-2. Twenty-seven of the initial 36 convalescent patients were monitored over the complete 9-month period following the onset of symptoms. The percentages of S-1, S-2, M and N peptide pool-specific T cells decreased only slightly over the 9-month period post infection (**Figures 4A–D**). Comparable results were also obtained when analyzing the frequencies of CD154^+^/IFN-γ^+^, CD154^+^/IL-2^+^ or CD154^+^/TNF-α^+^ CD4^+^ T cells. The amount of S-2-reactive CD154^+^/IFN-γ^+^ cells decreased significantly between V1 and V3. Similarly, the amount of either N-reactive CD154^+^/IFN-γ^+^ or CD154^+^/IL-2^+^ CD4^+^ T cells also decreased significantly between V2 and V3 (**Figure S6**). Breaking down the T cell responses of each individual against the SARS-CoV-2 peptide pools revealed that there are some donor-specific fluctuations in T cell frequency. In some donors the frequency of reactive T cells increased with each consecutive visit whereas it waned over time in others (**Figure S7**). The differences between the percentages of reactive T cells obtained from unexposed and convalescent groups remained similar (**Figure S8**).

Focusing on the proportional response rates, antigen recognition increased slightly between 3 and 6 months then decreased by 9 months (**Figure 4E**). The number of subjects that recognize at least one SARS-CoV-2 peptide pool increased from 88.9% to 100% and then decreased to 74% after 9 months. Similar trajectories were observed when analyzing the responses to the individual pools. The response to the N peptide pool was unique, inducing a CD4^+^ T cell response in 70.4% of convalescents at 3 months post symptom onset, which increased to 92.3% after 6 months, then declined to 55.6% at 9 months, a value comparable to the response to the spike and membrane proteins. There was also a slight shift in the number of peptide pools recognized as the time post symptom onset increased (**Figure 4F**). Initially, 41% of patients responded to four peptide pools. After 9 months, this response rate fell to 35%. At the same time, the number of subjects who recognized only one pool increased by about 10%. The number of patients recognizing two or three peptide pools peaked after 6 months and then decreased thereafter.

**Figure 5:**
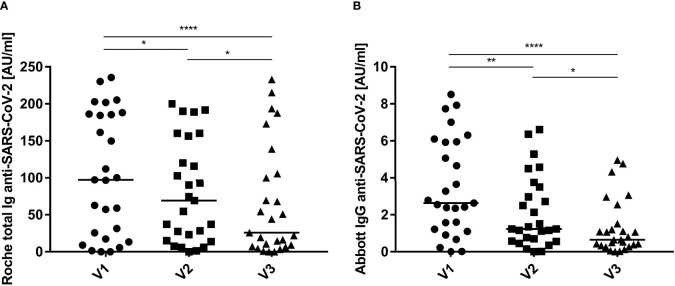
Humoral immunity to SARS-CoV-2 expressed over time. Total **(A**, Roche**)** and IgG-specific **(B**, Abbott**)** anti-SARS-CoV-2 immunoglobulin titers were quantified in the serum of convalescent individuals at: 3 months (V1, circles), 6 months (V2, squares) and 9 months (V3, triangles) post infection and symptom onset. Data are shown with medians (n = 27). Statistically different: *P < 0.05, **P < 0.01, ****P < 0.0001 (Friedman test).

To compare cellular and humoral immunity to SARS-CoV-2 post symptom onset over time, total and IgG-specific anti-SARS-CoV-2 immunoglobulin titers in convalescent donors were monitored using the Roche and Abbott serologic assays, respectively (**Figure 5**). Antibody titers displayed a wide range, but typically decreased regardless of the serologic assay used for analysis. In general, antibody titers started to decrease with each consecutive time point. The median antibody levels decreased ~75% over the course of the 9-month study according to the results of both assays. Breaking down antibody titers for each individual donor revealed similar results, i.e., titers decreased with each consecutive visit in most donors (**Figure S9**).

## Discussion

The emergence of COVID-19 at the end of 2019 has exerted a global impact on mankind. A better understanding of the factors affecting the immune response to SARS-CoV-2 infections, the longevity of immunity and the effectivity of vaccines are vital issues facing society today. Previous reports clearly showed that SARS-CoV-2 infections induce virus-specific cellular and humoral immune responses (20–23). T cells and serum antibodies specific for a number of virus structural proteins were readily detected in convalescent patients (6, 21, 22). Compared to unexposed individuals, convalescent patients exhibited greater CD4^+^ T cell reactivity toward peptide pools derived from the SARS-CoV-2 structural proteins S, M and N at approximately 3 months post infection. Those individuals showed increased percentages of specific CD4^+^ T cells, and reacted in much greater frequency to peptide pools derived from more than one structural protein. Indeed, Grifoni and co-workers also reported as well that convalescent subjects reacted to peptide pools derived from different structural proteins (8). Other investigators reported frequencies ranging from 60 to 80% of reactive individuals (23–25). The frequency of individuals who responded to a peptide pool derived from any single structural protein in the study reported herein was approximately the same.

Interestingly, T cells specific for SARS-CoV-2-related peptide pools were also detected in a sizable number of unexposed individuals. Forty-seven percent of these individuals recognized at least one peptide pool; 28% recognized the spike glycoprotein S-2 pool most often. Similar numbers were reported by Braun and colleagues who measured specific T cells against the S-2 peptide pool in 35% of unexposed, healthy individuals (23). Several other groups reported comparable numbers of 20-50% of unexposed, healthy individuals reacting against SARS-CoV-2 (6, 8, 20, 26, 27). Furthermore, Braun et al. showed that T cells derived from these unexposed individuals reacted toward common coronavirus strains (23). Indeed, comparing unexposed and convalescent subjects in the present study, little difference was found in their reaction toward endemic coronaviruses assessed in terms of cell number and frequency of response. Bonifacius et al. reported similar results, i.e., no difference in the responses of unexposed and convalescent study participants (25). Only active COVID-19 patients showed lower frequencies of HCoV-reactive T cells.

Immunity against seasonal coronaviruses tends to be short-lived as it is not uncommon to be infected with the same virus strain every 12 months (28, 29). Nevertheless, in a study performed by Gorse et al. 90-100% of subjects were seropositive for the four most common HCoV strains (30). There are conflicting reports, however, concerning the ability of a preceding HCoV infection to confer SARS-CoV-2 protection. Reportedly, SARS-CoV-2 patients capable of producing anti-OC43 antibodies did not develop severe pneumonia (31). Similarly, Loyal et al. were able to show that pre-existing spike-cross-reactive T cells were activated after COVID-19 mRNA vaccination and showed signs similar to a secondary immune response (32). Contrasting studies that measured a high degree of cross-reactivity between SARS-CoV-2 and HCoV, however, found no correlation between HCoV reactivity and increased COVID-19 immunity (33, 34). This is in line with other studies showing that HCoV-specific antibody titers increased in convalescent individuals but did not correlate with increased SARS-CoV-2 titers or protection (35–37). In the study reported here, increased numbers of SARS-CoV-2-reactive T cells were detected in individuals who were also HCoV-reactive. Conversely, HCoV-reactive individuals tended to react more frequently to the peptide pools derived from SARS-CoV-2 structural proteins. This increase in frequency was also noted in the cohort not exposed to SARS-CoV-2. Interestingly, this finding is in contrast to a study performed by Woldemeskel et al. in which only 1 of 21 tested healthy donors showed a response against SARS-CoV-2 spike or nucleocapsid protein (38). This effect was more pronounced for C-terminal peptide pools derived from spike. This could be due to greater homology between the C-terminuses, than the N-terminuses, of the spike proteins of SARS-CoV-2 and endemic human coronaviruses (23). Nevertheless, it is not possible to conclude from the present study those individuals previously infected with endemic coronaviruses were better protected from severe COVID-19 outcomes since all convalescent individuals enrolled in the current study were either asymptomatic or experienced only mild disease.

Persistence of immune memory to SARS-CoV-2 infection was a central issue of the current study. A cohort of convalescent healthcare workers studied over a 9-month period following infection and symptom onset demonstrated a stable cellular immune response. This finding correlates with the results of other studies (13, 14, 21, 24). Notably, most studies including the one described here focused primarily on mild and asymptomatic COVID-19 cases; conflicting reports exist regarding immune persistence in hospitalized patients (39, 40).

In contrast to a relatively stable T cell response, sharp declining SARS-CoV-2-specific antibody titers were found over the 9-month period following infection and symptom onset. This decline in antibody titers agrees with the reports of other investigators, and is apparently not related to the response to a specific viral protein or region (41–43). Cohen and coworkers calculated the half-life of serum IgG specific for the spike glycoprotein, spike receptor binding domain or the spike N-terminal domain was ~120 days, roughly equivalent to the decline determined herein for the median SARS-CoV-2-specific antibody titers (24). Taken together, these finding emphasize the import of considering T cell responses, rather than antibody titers, as a measure of SARS-CoV-2 protection. Notably, ~10% of SARS-CoV-2-infected patients did not produce IgG following recovery although circulating, virus-specific T cells were detected (44–46).

The SARS-CoV-2 nucleocapsid protein induced the greatest CD4^+^ T cell response by the convalescent cohort in the study reported herein. Indeed, N induced the biggest increase in T cell reactivity between the third- and sixth-months post infection. Other studies report increased anti-spike glycoprotein T cell reactivity with the passage of time post infection (22, 24, 25). In this regard, it is relevant that all approved COVID-19 mRNA vaccines focus solely upon spike glycoprotein sequences (17, 18). However, in light of the current study, it seems reasonable to explore the construction of new vaccines that target the nucleocapsid as well, and thus improve vaccine efficacy. The nucleocapsids of SARS-CoV-2 and other betacoronaviruses exhibit a high degree of homology (20, 47). The N protein should be less prone to mutation since it is not under the same selective pressure as the spike receptor binding domain. SARS-CoV-2 variants have already demonstrated resistance to neutralizing antibodies (48, 49). Le Bert et al. reported that long-term memory T cells obtained from SARS-CoV patients 17 years after the outbreak of SARS still reacted to the N protein of SARS-CoV (20). Interestingly, they also observed these T cells cross-reacted with the N protein of SARS-CoV-2; notably, the response to other SARS-CoV-2 structural proteins was not examined.

In summary, new evidence is presented that documents the degree of cross-reactivity between SARS-CoV-2 and HCoV in SARS-CoV-2-convalescent, as well as unexposed, individuals. It is not clear, though, whether prior immunity to HCoV increases protection against SARS-CoV-2. Cellular immunity acquired as a consequence of SARS-CoV-2 infection remains stable for up to 9 months. Determining whether SARS-CoV-2-specific T cell reactivity persists for a longer period will require additional studies. Humoral immunity and SARS-CoV-2-specific antibody titers, on the other hand, decline fairly rapidly over the course of a 9-month period following infection. As such, T cell reactivity may be a much more suitable correlate of protective immunity. Moreover, the data indicate that incorporating sequences that encode structural SARS-CoV-2 protein sequences in addition to the spike glycoprotein could improve the efficacy of next generation COVID-19 mRNA vaccines.

## Data Availability Statement

The datasets generated and/or analysed during the current study are available from the corresponding author on reasonable request.

## Ethics Statement

The studies involving human participants were reviewed and approved by Ethikkommision der Landesärztekammer Rheinland-Pfalz. The patients/participants provided their written informed consent to participate in this study.

## Author Contributions

SW, LH, MH, CM, and SG designed the project. SW, LH, MH, BG, and KH performed the experiments. SW, LH, and MH analyzed the data. SW, LH, MH, FK, CM, and SG discussed the data. SW wrote the manuscript with the contribution of all other co-authors. All authors contributed to the article and approved the submitted version.

## Conflict of Interest Statement

The authors declare that the research was conducted in the absence of any commercial or financial relationships that could be construed as a potential conflict of interest.

## Publisher’s Note

All claims expressed in this article are solely those of the authors and do not necessarily represent those of their affiliated organizations, or those of the publisher, the editors and the reviewers. Any product that may be evaluated in this article, or claim that may be made by its manufacturer, is not guaranteed or endorsed by the publisher.
