# Supplementary material for: Long-Term, CD4+ Memory T Cell Response to SARS-CoV-2
Source: Front Immunol. 2022 Apr 20;13:800070. doi: 10.3389/fimmu.2022.800070 (PMC9065554; doi:10.3389/fimmu.2022.800070)
Supplement: Supplementary file 1 [file DataSheet_1.docx]

**Supplementary Material**

**Supplementary Table 1: Study cohorts**

|  | **Unexposed (n=36)** | **Convalescent (n=36)** |
| --- | --- | --- |
| **Age** | 37.1 | 35.8 |
| <30 years | 33.3% (n=12) | 44.4% (n=16) |
| 30-39 years | 27.8% (n=10) | 19.4% (n=7) |
| 40-49 years | 25.0% (n=9) | 22.2% (n=8) |
| 50-59 years | 8.3% (n=3) | 8.3% (n=3) |
| >60 years | 5.6% (n=2) | 5.6% (n=2) |
| **Sex** |  |  |
| Male | 30.6% (n=11) | 30.6% (n=11) |
| Female | 69.4% (n=25) | 69.4% (n=25) |
| **Pre-existing condition** |  |  |
| Hypertension | 2.8% (n=1) | 2.8% (n=1) |
| Asthma | 2.8% (n=1) | 2.8% (n=1) |
| Nicotine abuse | 19.4% (n=7) | 30.6% (n=11) |
| **Weight (BMI)** |  |  |
| Mean | 26.2 kg/m^2^ | 26.25 kg/m^2^ |
| Median | 24.65 kg/m^2^ | 24.9 kg/m^2^ |
| BMI >30 | 13.89% (n=5) | 22.22% (n=8) |
| **SARS-CoV-2 test** |  |  |
| PCR | 0% | 63.9% (n=23) |
| Serological | 0% | 94.4% (n=34) |
| **Symptoms** |  |  |
| Fever | 0% (n=0) | 41.67% (n=15) |
| Dry cough | 16.67% (n=6) | 44.44% (n=16) |
| Loss of smell/taste | 2.78% (n=1) | 63.89% (n=23) |
| Diarrhea | 8.33% (n=3) | 25.00% (n=9) |
| Rhinitis | 11.11% (n=4) | 27.78% n= (10) |
| Muscle pain | 13.89% (n=5) | 47.22% (n=17) |
| Headache | 44.44% (n=16) | 63.89% (n=23) |


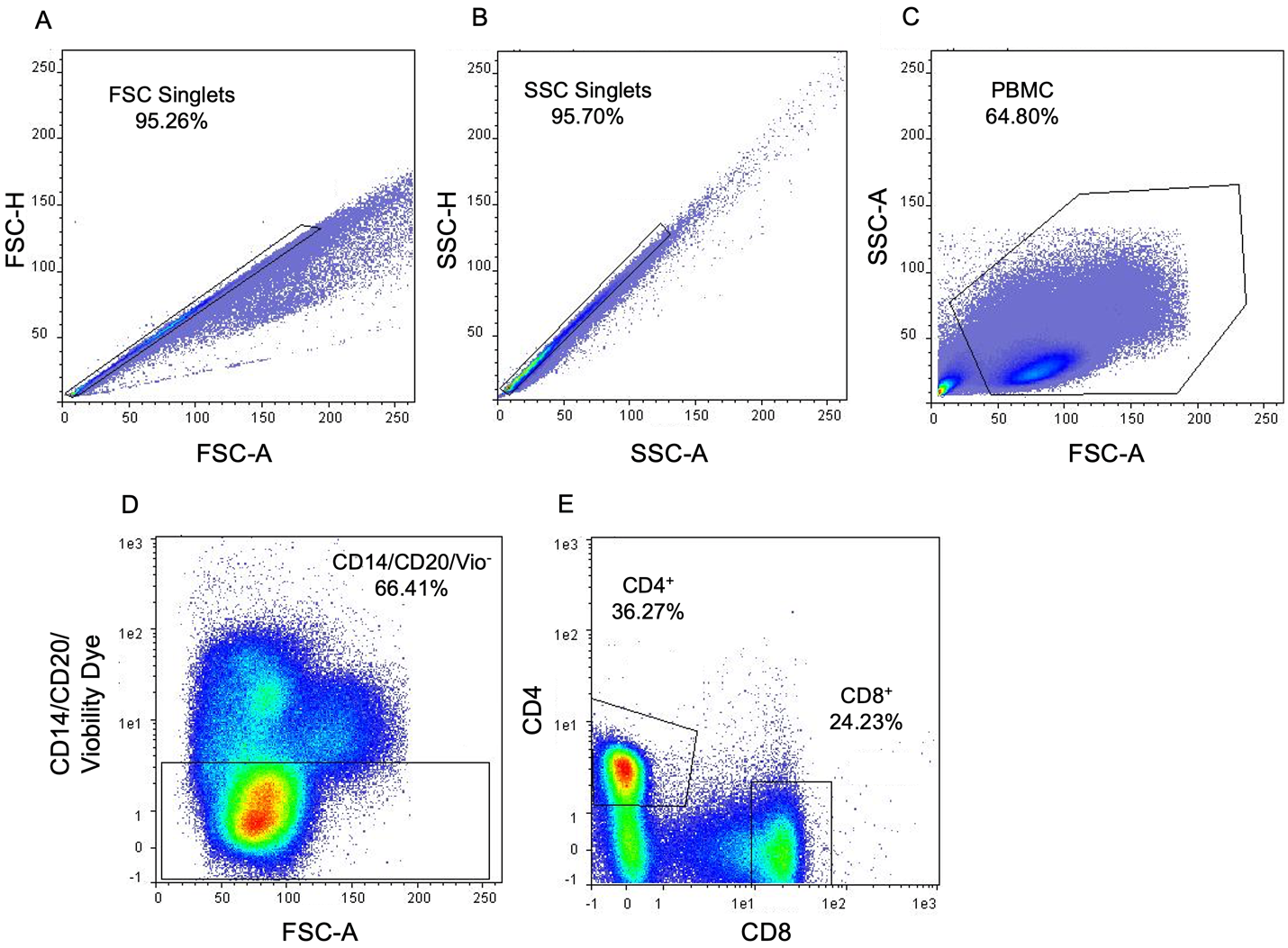


**Supplementary Figure 1: Gating strategy to define CD4^+^ T cells.** A CD4^+^ T cell population was defined by excluding forward scatter (A) and sidewards scatter (B) doublets before gating on the PBMC population (C). Next, CD14^+^, CD20^+^ and Viobility Dye^+^ cells were excluded (D). Cells were then differentiated into CD4^+^ and CD8^+^ T cells (E).


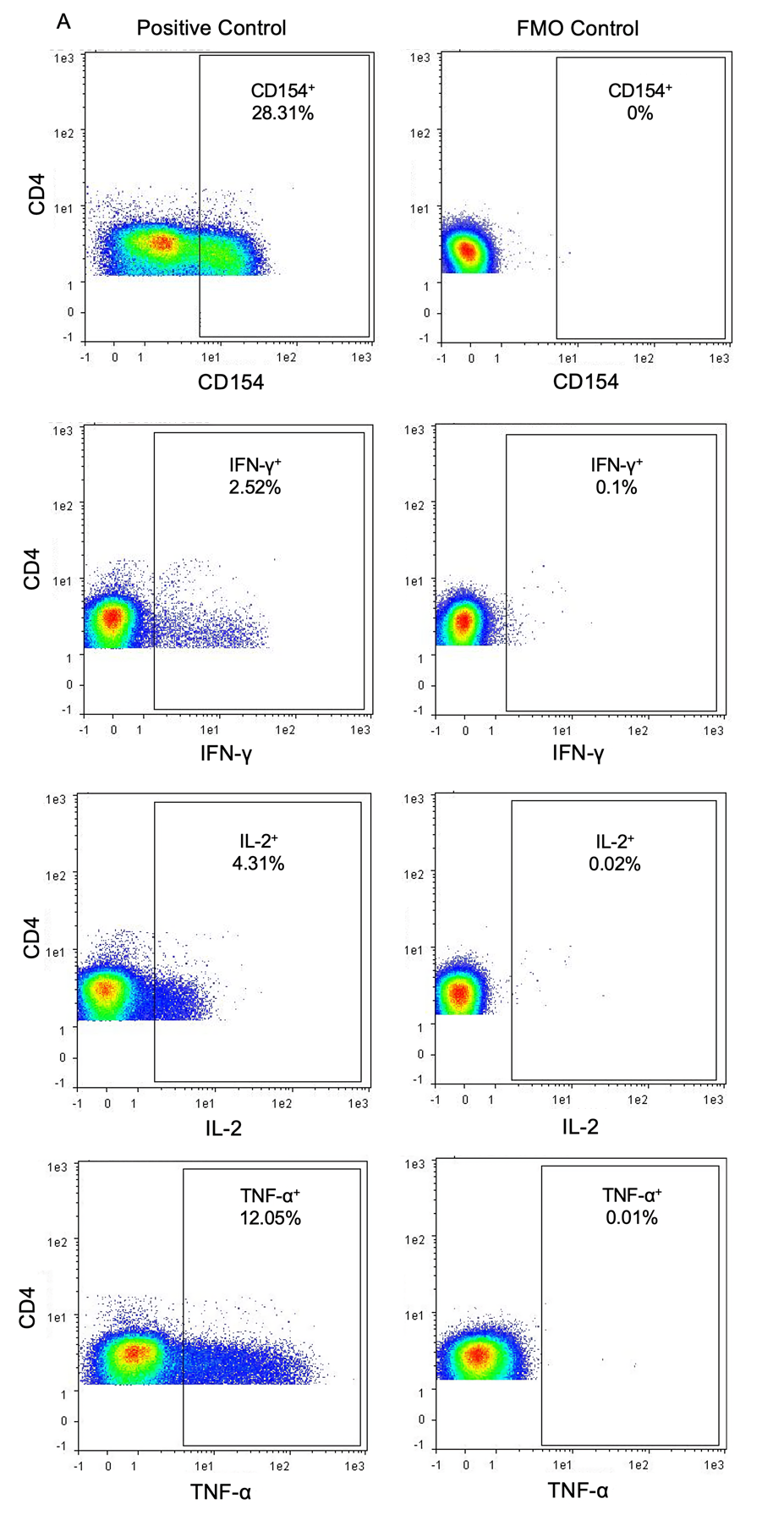


**Supplementary Figure 2: Gating strategy to define SARS-CoV-2-reactive CD4^+^ T cells.** Reactive CD4^+^ T cells were selected based on their expression of the 4 activation markers CD154 (A), IFN-γ (B), IL-2 (C) and TNF-α (D). Positive populations were defined based on PBMCs stimulated with SEB (1.5 µg/ml) (left side) and a corresponding FMO control (right side).


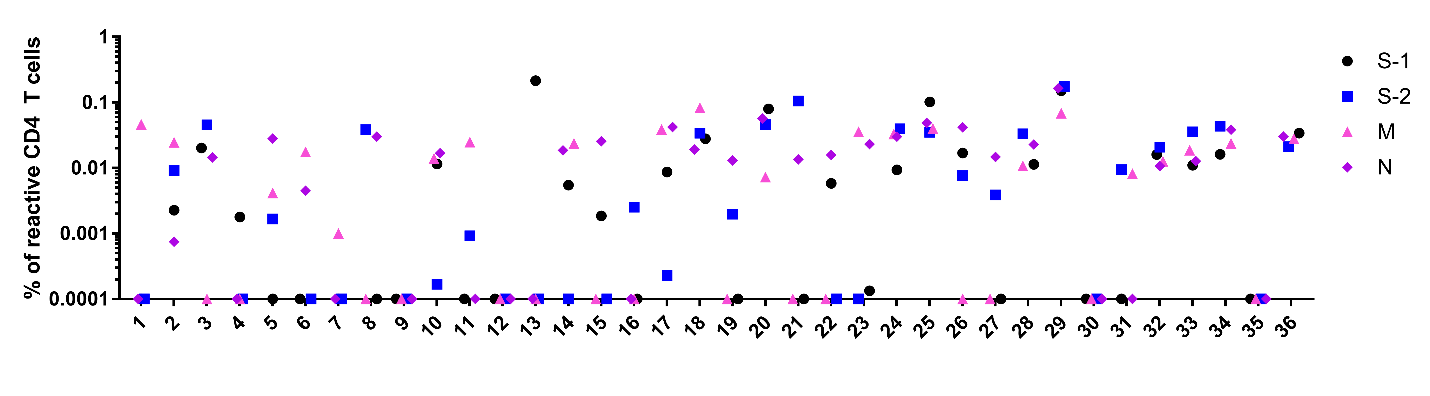


**Supplementary Figure 3: CD4^+^ T cell responses of individual donors against SARS-CoV-2 peptide pools.** T cell responses of each individual convalescent donor against S-1 (black circles), S-2 (blue squares), M (pink triangles) and N (purple rhombuses) SARS-CoV-2 peptide pools. Reactive T cells were defined as CD4^+^ T cells expressing >2 T_H1_ activation markers (CD154, IFN-γ, IL-2, TNF-α). DMSO background controls were subtracted from the data shown.


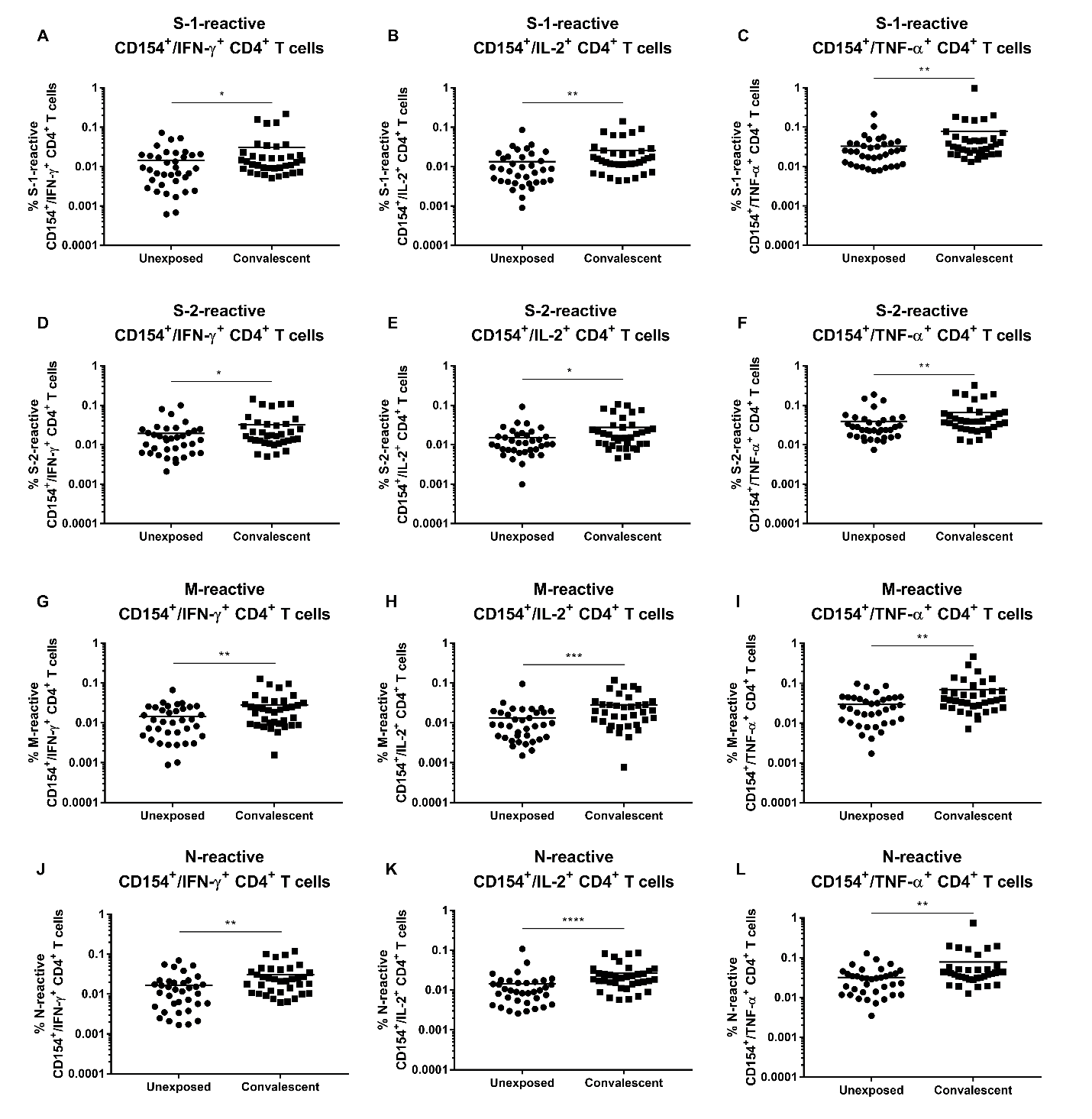


**Supplementary Figure 4: CD4^+^ T cell response specific for SARS-CoV-2.** T cell responses of unexposed (circles) and convalescent (squares) individuals against S-1 (A-C), S-2 (D-F), M (G-I) or N (J-L) SARS-CoV-2 peptide pools. Analysis of CD154^+^/IFN-γ^+^ (A, D, G, J), CD154^+^/IL-2^+^ (B, E, H, K) and CD154^+^/TNF-α^+^ (C, F, I, L) SARS-CoV-2-reactive CD4^+^ T cells. DMSO background controls were subtracted from the data shown. Data are shown with the means (n = 36). Statistically different: **P* <0.05, ***P* <0.01, ****P* <0.005 (non-parametric two-tailed Mann-Whitney-U test).

**
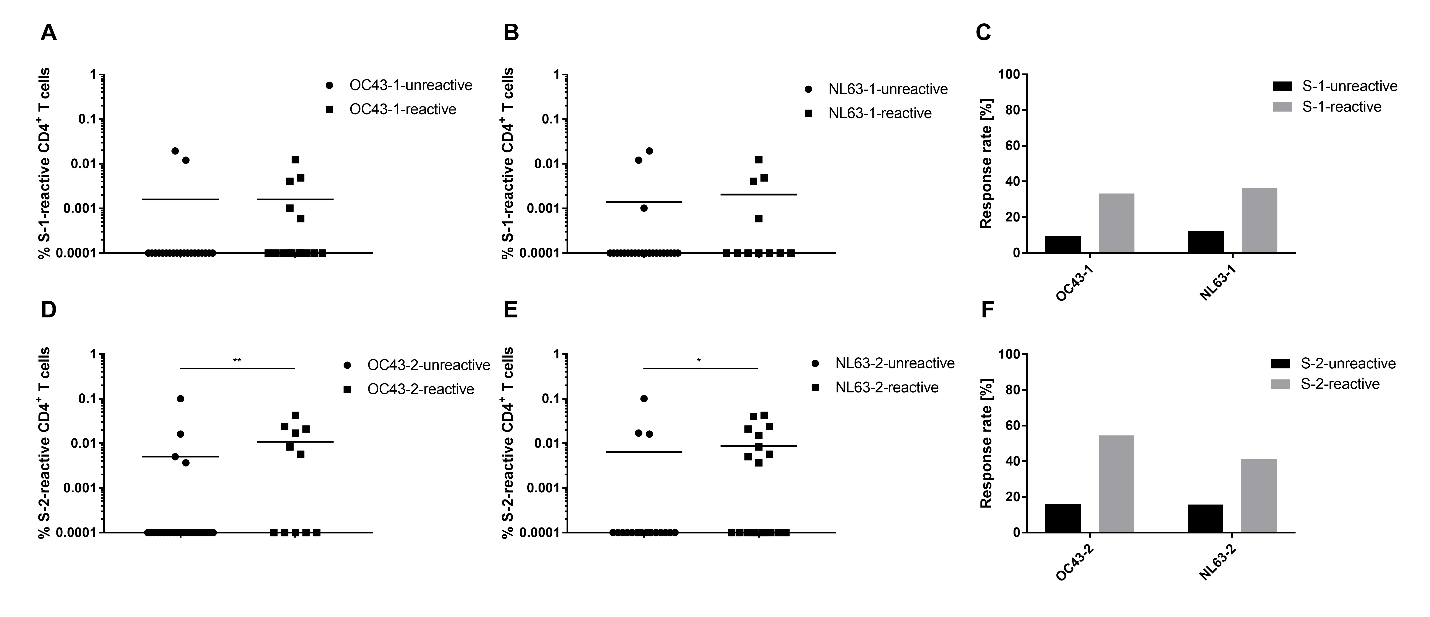
**

**Supplementary Figure 5: Analysis of SARS-CoV-2 cross-reactivity with endemic HCoV antigens in unexposed individuals.** Unexposed individuals unreactive (circles) or reactive (squares) against OC43 and NL63 N-terminal (A, B) or C-terminal (D, E) HCoV peptide pools were analyzed for their responses to their respective SARS-CoV-2 counterpart. (C, F) Comparison of the relative response rate of HCoV unreactive (black) and reactive (grey) individuals against SARS-CoV-2 S-1 (C) and S-2 (F) antigen. Reactive T cells were defined as CD4^+^ T cells expressing >2 T_H1_ markers (CD154, IFN-γ, IL-2, TNF-α). DMSO background controls were subtracted from the data shown. Data are the means. (A) unreactive n = 21; reactive n = 15, (B) unreactive n = 25; reactive n = 11, (D) unreactive n = 25; reactive n = 11, (E) unreactive n = 21; reactive n = 19. Statistically different: **P* <0.05 (non-parametric two-tailed Mann-Whitney-U test).


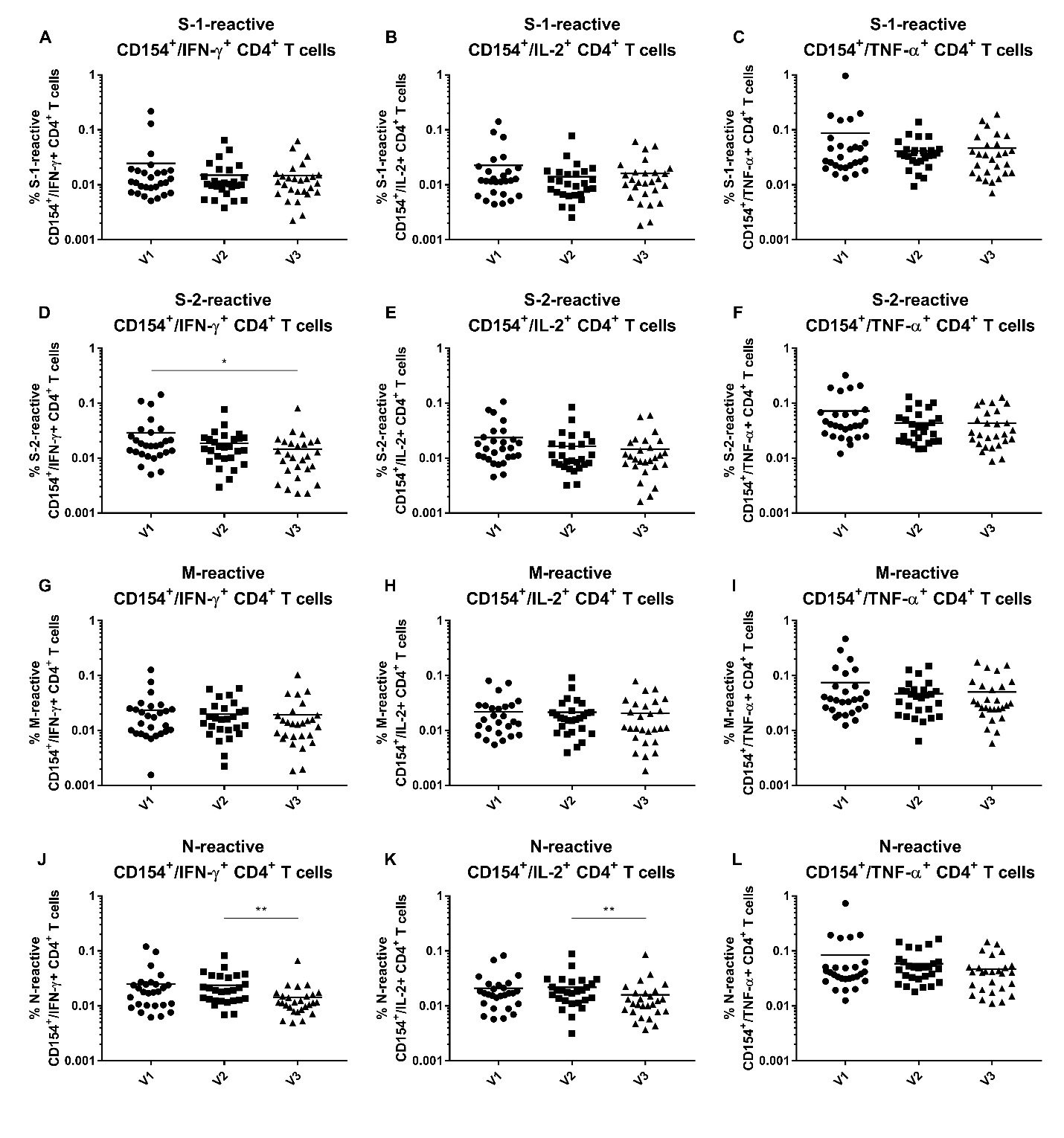


**Supplementary Figure 6: Development of peptide-specific CD4^+^ T cell responses.** Comparison of immune responses of convalescent individuals against S-1 (A-C), S-2 (D-F), M (G-I) and N (J-L) SARS-CoV-2 peptide pools over the course of a 9-month period. Peripheral blood was drawn at three timepoints: 3 months (V1, circles), 6 months (V2, squares) and 9 months (V3, triangles) post infection and symptom onset. Analysis of CD154^+^/IFN-γ^+^ (A, D, G, J), CD154^+^/IL-2^+^ (B, E, H, K) and CD154^+^/TNF-α^+^ (C, F, I, L) SARS-CoV-2-reactive CD4^+^ T cells. DMSO background controls were subtracted from the data shown. Data are shown with the means (n = 36). Statistically different: **P* <0.05, ***P* <0.01, ****P* <0.005 (non-parametric two-tailed Mann-Whitney-U test).

**
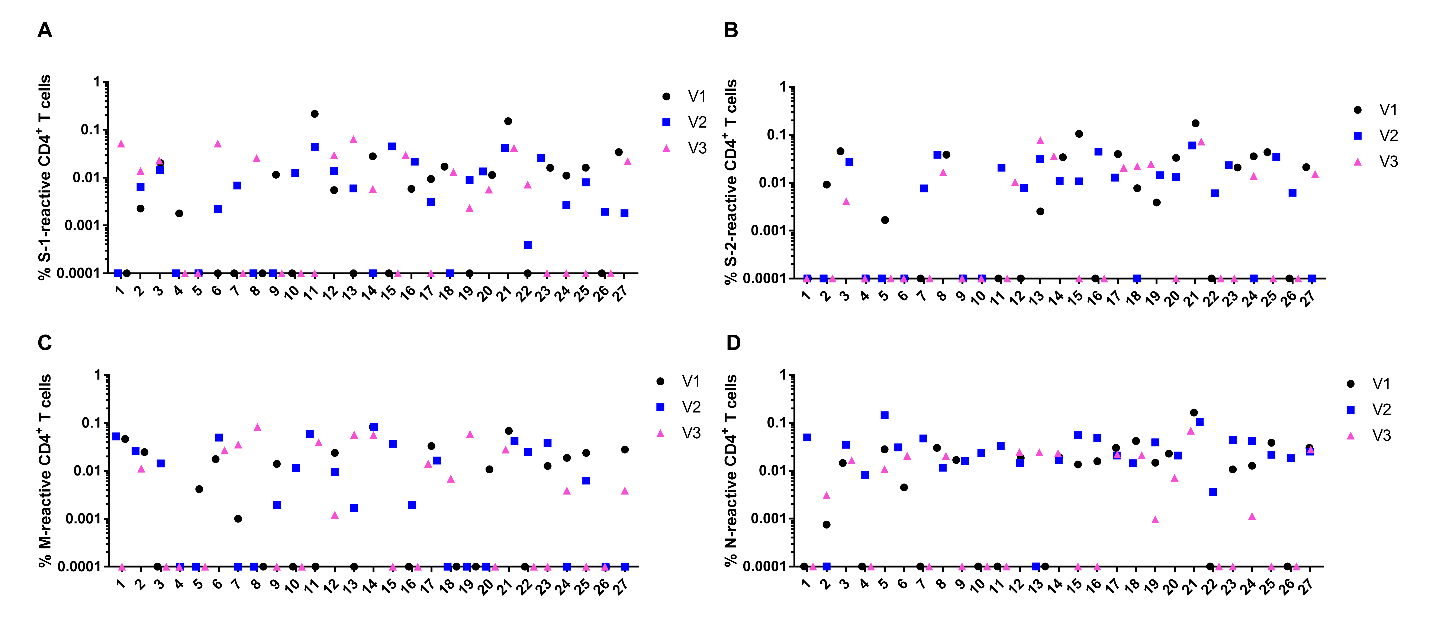
**

**Supplementary Figure 7: Development of peptide-specific CD4^+^ T cell responses of individual convalescent donors.** Comparison of immune responses of individual convalescent donors against S-1 (A), S-2 (B), M (C) and N (D) SARS-CoV-2 peptide pools over the course of a 9-month period. Peripheral blood was drawn at three timepoints: 3 months (V1, black circles), 6 months (V2, blue squares) and 9 months (V3, pink triangles) post infection and symptom onset. Reactive T cells were defined as CD4^+^ T cells expressing >2 T_H1_ markers (CD154, IFN-γ, IL-2, TNF-α). DMSO background controls were subtracted from the data shown.


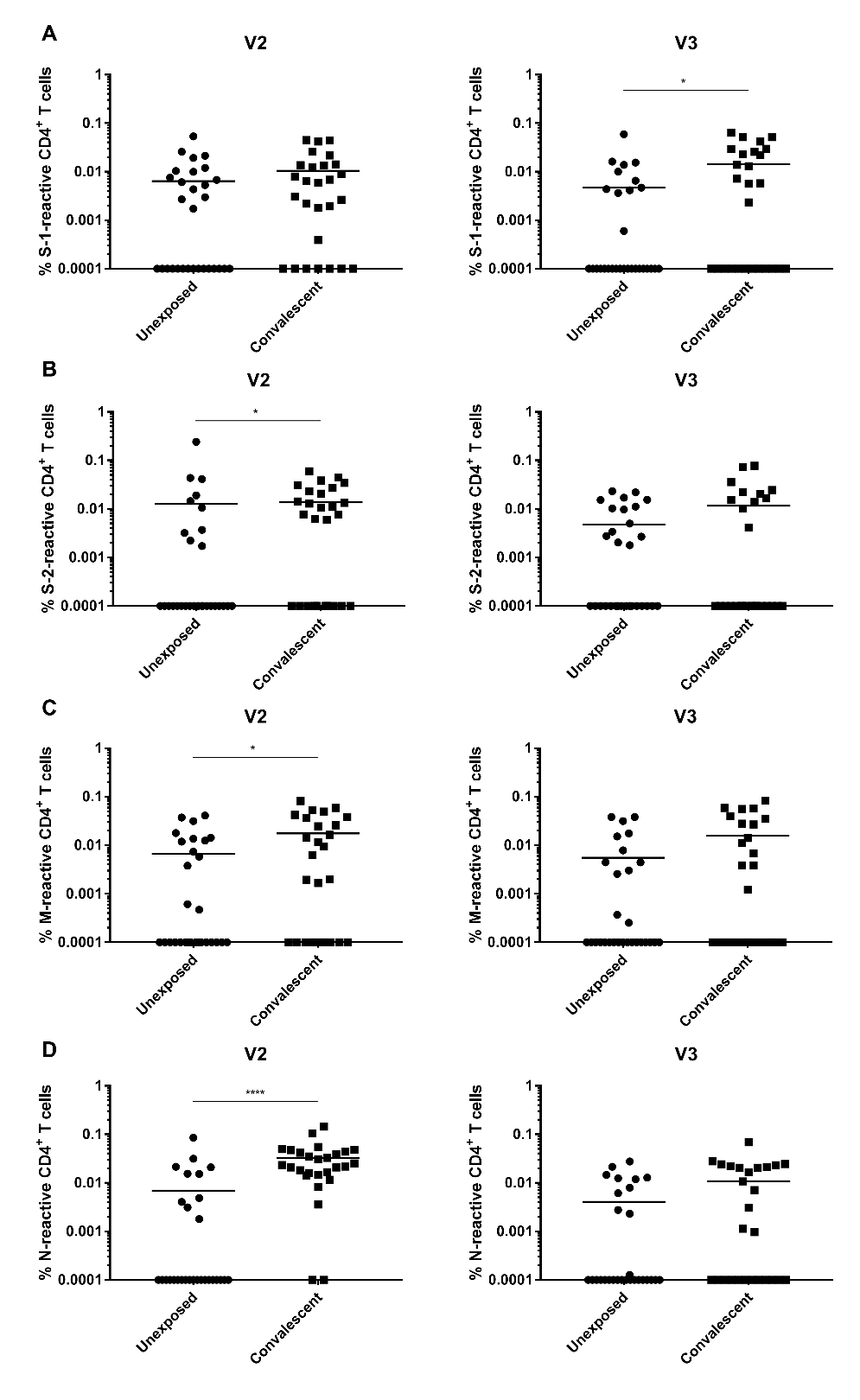


**Supplementary Figure 8: Comparison of CD4^+^ T cell response by unexposed and convalescent individuals at approximate 6- and 9-months post infection.** T cell responses of unexposed (circles) and convalescent (squares) individuals to S-1 (A), S-2 (B), M (C) or N (D) SARS-CoV-2 peptide pools. Results shown are: 6 months (V2, left side) and nine months (V3, right side) post infection. Reactive T cells were defined as CD4^+^ T cells expressing >2 T_H1_ markers (CD154, IFN-γ, IL-2, TNF-α). DMSO background controls were subtracted from the data shown. Data are shown with means. Unexposed n = 30; Convalescent n = 27. Statistically different: **P* <0.05, *****P* <0.0001 (non-parametric two-tailed Mann-Whitney-U test).


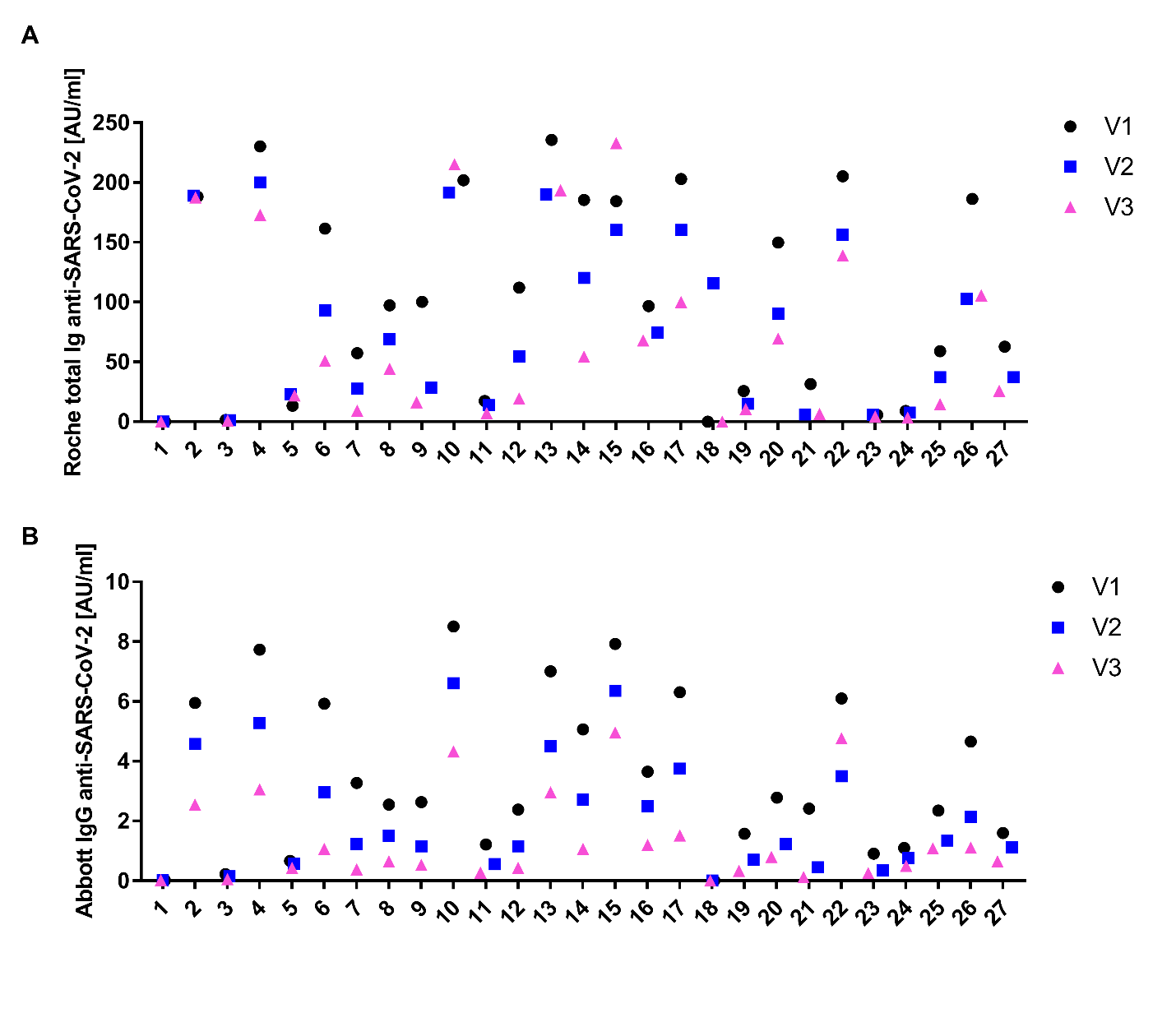
**Supplementary Figure 9: Humoral immunity to SARS-CoV-2 of individual convalescent donors expressed over time.** Total (A, Roche) and IgG-specific (B, Abbott) anti-SARS-CoV-2 immunoglobulin titers were quantified in the serum of individual convalescent donors at: 3 months (V1, black circles), 6 months (V2, blue squares) and 9 months (V3, pink triangles) post infection and symptom onset.
